# Liquid biopsies to predict CDK4/6 inhibitor efficacy and resistance in breast cancer

**DOI:** 10.20517/cdr.2022.37

**Published:** 2022-06-22

**Authors:** Sasha C. Main, David W. Cescon, Scott V. Bratman

**Affiliations:** ^1^Princess Margaret Cancer Centre, University Health Network, Toronto M5G 2C1, Ontario, Canada.; ^2^Department of Medical Biophysics, University of Toronto, Toronto M5G 1L7, Ontario, Canada.; ^3^Division of Medical Oncology and Hematology, Department of Medicine, University of Toronto, Toronto M5S 1A8, Ontario, Canada.; ^4^Department of Radiation Oncology, University of Toronto, Toronto M5T 1P5, Ontario, Canada.

**Keywords:** Breast cancer, liquid biopsy, circulating tumor DNA, cell-free DNA, CDK4/6 inhibitors, resistance mechanisms, predictive biomarkers, circulating biomarkers

## Abstract

Cyclin-dependent kinase 4 and 6 (CDK4/6) inhibitors combined with endocrine therapy have transformed the treatment of estrogen receptor-positive (ER+) and human epidermal growth factor receptor 2 negative (HER2-) metastatic breast cancer. However, some patients do not respond to this treatment, and patients inevitably develop resistance, such that novel biomarkers are needed to predict primary resistance, monitor treatment response for acquired resistance, and personalize treatment strategies. Circumventing the spatial and temporal limitations of tissue biopsy, newly developed liquid biopsy approaches have the potential to uncover biomarkers that can predict CDK4/6 inhibitor efficacy and resistance in breast cancer patients through a simple blood test. Studies on circulating tumor DNA (ctDNA)-based liquid biopsy biomarkers of CDK4/6 inhibitor resistance have focused primarily on genomic alterations and have failed thus far to identify clear and clinically validated predictive biomarkers, but emerging epigenetic ctDNA methodologies hold promise for further discovery. The present review outlines recent advances and future directions in ctDNA-based biomarkers of CDK4/6 inhibitor treatment response.

## INTRODUCTION

Breast cancer is the most diagnosed cancer in women globally, with approximately 70% of diagnoses being tumors that express estrogen receptors (ER+) but not human epidermal growth factor receptor 2 (HER2-)^[1]^. In jurisdictions that have implemented breast cancer screening programs, many ER+/HER2- breast cancers are diagnosed in localized or locoregional stages and are amenable to curative intent therapy. However, despite multimodality treatments, patients have a lifelong risk of metastatic recurrence, and once distant metastasis presents clinically, it is typically incurable^[2,3]^.

In the past decade, novel therapeutic strategies for metastatic breast cancer patients have been implemented in the clinic. Among these, the new standard treatment for ER+/HER2- locally advanced and metastatic breast cancer consists of cyclin-dependent kinase 4 and 6 (CDK4/6) inhibitors combined with endocrine therapy^[4]^. Three CDK4/6 inhibitors have been approved (i.e., abemaciclib, palbociclib, and ribociclib) after displaying significant clinical benefit in pivotal phase III clinical trials^[5-12]^. CDK4/6 inhibitors have been shown to improve response rate, median progression-free survival (PFS), health-related quality of life, and overall survival (OS) of metastatic breast cancer patients^[13-19]^. However, CDK4/6 inhibitor resistance remains a significant obstacle. A minority of patients have intrinsic resistance, defined as progression (without response) within six months of starting treatment. Even for patients who experience initial response and clinical benefit from these agents, acquired resistance inevitably develops over subsequent months (median PFS ranges from 23.8-28.2 months in the first-line metastatic setting)^[11,17,20]^. Therefore, biomarkers are urgently needed to predict CDK4/6 inhibitor efficacy or resistance in metastatic breast cancer patients, allowing clinicians to tailor treatment and potentially add additional therapies for patients at high risk of early progression.

Biomarkers for CDK4/6 inhibitors have been thoroughly investigated through molecular profiling of tumor material, but to date, the only clinically available biomarker remains breast cancer subtype as defined by traditional tissue markers (i.e., ER+/HER2-)^[21,22]^. Despite significant research efforts, tumor heterogeneity and difficulties distinguishing endocrine resistance from CDK4/6 inhibitor resistance have impeded predictive biomarker discovery^[23]^. Given practical challenges to obtaining and repeating metastatic tissue biopsies, blood-based profiling of tumor-derived material (i.e., “liquid biopsy”) has significant potential to facilitate biomarker explorations. For instance, circulating tumor cells (CTCs) and circulating tumor DNA (ctDNA) are promising liquid biopsy analytes because they harbor cancer-specific molecular aberrations^[24]^.

In this review, we discuss biomarker-directed treatment for breast cancer, mechanisms of resistance to CDK4/6 inhibitors, and attempts to uncover ctDNA liquid biopsy biomarkers of efficacy and resistance to CDK4/6 inhibitors. Lastly, we highlight undeveloped areas for future advances, namely epigenetic-based liquid biopsy biomarkers for patients treated with CDK4/6 inhibitors.

### BIOMARKER-DIRECTED PRECISION ONCOLOGY AND BREAST CANCER

Precision oncology relies on molecular information from each patient’s cancer to optimize and individualize treatment regimens. Leveraging these patient-specific molecular biomarkers allows clinicians to select the best treatment for individual patients to improve therapeutic efficacy and reduce adverse effects on healthy cells^[25]^. Molecular biomarkers include prognostic biomarkers, which may provide information on the expected disease course independent of treatment, and predictive biomarkers, which provide insight into the effect of a specific therapy. Both prognostic and predictive biomarkers may be used to personalize treatment via risk-stratification or directing effective treatments.

Current standard-of-care breast cancer treatments provide several archetypal examples of biomarker-directed precision oncology. For instance, OncotypeDx is a commercial 21-gene assay for ER+ early-stage breast cancer that returns a recurrence score indicating the probability of relapse without adjuvant chemotherapy, with higher scores associated with a poorer prognosis. OncotypeDx serves a prognostic role; its clinical utility stems from identifying patients with a higher absolute recurrence risk and, therefore, a higher likelihood of benefiting from adjuvant chemotherapy^[26,27]^. Similarly, the MammaPrint microarray assay is a prognostic biomarker that uses the expression levels of 70 genes to classify patients according to recurrence risk^[28,29]^.

Other breast cancer biomarkers highlight the impact of predictive biomarkers in precision oncology. Intrinsic molecular subtypes and associated hormone receptors (ER, PR) and HER2 expression levels are critical for drug selection in breast cancer patients^[30]^. For instance, HER2+ breast cancer preferentially responds to HER2-targeted agents, such as trastuzumab and trastuzumab emtansine (T-DM1)^[31]^. Likewise, hormone receptor expression denotes tumors that preferentially respond to endocrine therapy. Resistance to endocrine therapy can occur over time through a variety of mechanisms (e.g., genetic alterations of *ESR1*, increased activity of cyclin-dependent kinases (CDKs), and mitogen-signaling pathways such as PI3K and RAS, or a decrease in proteins that inhibit CDKs such as p16, p21, and p27), several of which converge on the cyclin D-CDK4/6 axis^[32]^. Therefore, simultaneous treatment with endocrine therapy and CDK4/6 inhibitors has emerged as a highly successful treatment paradigm for ER+/HER2- metastatic breast cancer.

## MOLECULAR MECHANISMS OF CDK4/6 INHIBITOR EFFICACY AND RESISTANCE

**Figure 1:**
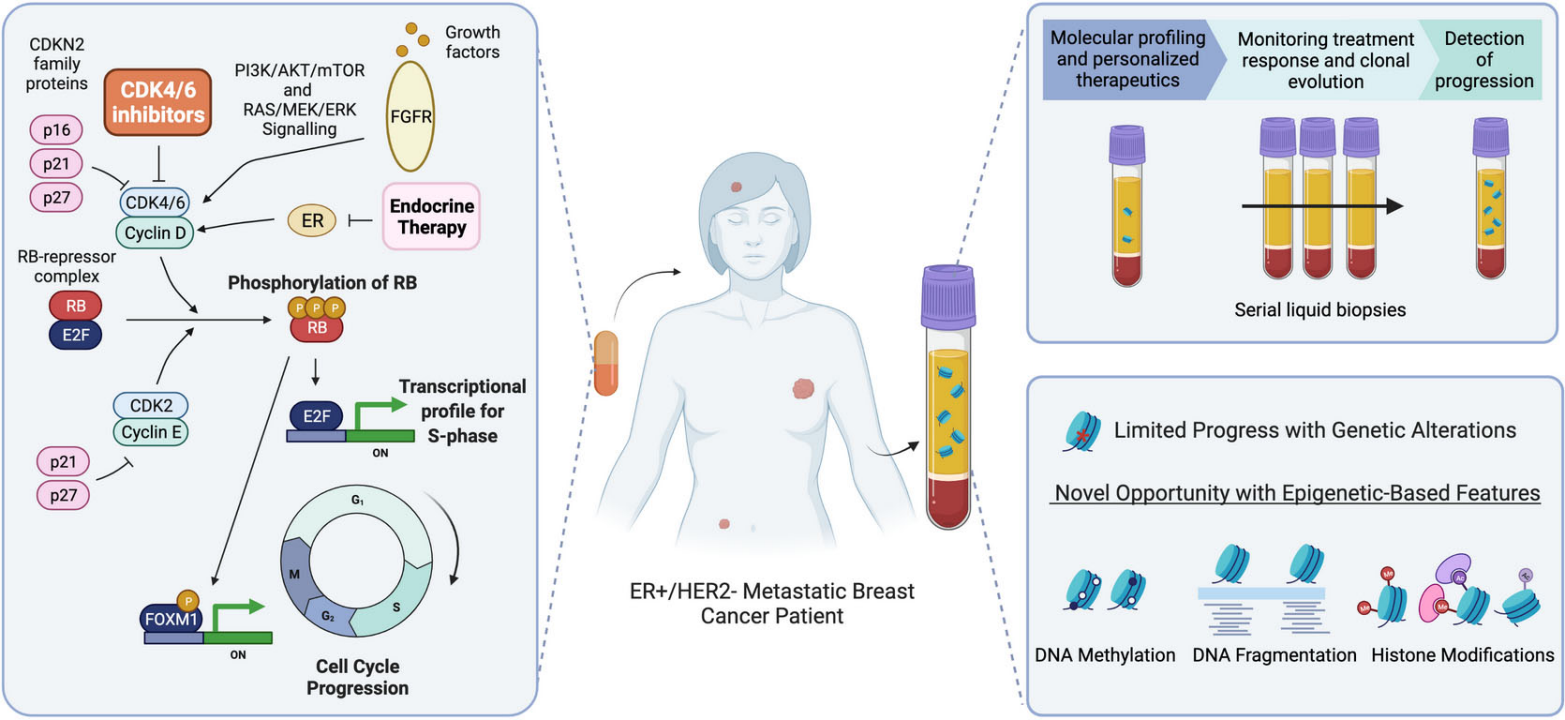
Overview of CDK4/6 inhibitor treatment and liquid biopsy in ER+/HER2- metastatic breast cancer patients. CDK4/6 inhibitors prevent the phosphorylation of Rb and downstream activation of the transcriptional profile required for progression to the S-phase of the cell cycle. In the anti-CDK4/6 therapy clinical setting, a liquid biopsy may be used to direct therapy, monitor patient response over time, and detect progression. Currently, research efforts focused on monitoring genetic alterations in cfDNA through liquid biopsy have made limited progress. Epigenetic profiling of cfDNA may reveal new biomarkers of CDK4/6 inhibitor efficacy or resistance.

The cyclin D-CDK4/6-Retinoblastoma protein (Rb) axis regulates cell cycle progression from G1 to the S phase [Figure 1]. Before entering the cell cycle, Rb is hypophosphorylated and bound to the E2F transcription factors (TFs), causing their inhibition^[33]^. When the appropriate mitogenic signals are present, quiescent cells may enter the cell cycle at the G1 phase. These mitogenic signals lead to the expression of cyclin D, which competes with CDKN2 family proteins to bind CDK4/6, forming the cyclin D-CDK4/6 complex^[34]^. This active complex can then phosphorylate Rb, causing a conformational change and subsequent release of the E2F TFs, which drive S phase entry and further cell cycle progression via downstream transcriptional activation^[33]^. Furthermore, the cyclin D-CDK4/6 complex triggers the forkhead box protein M1 (FOXM1) TF, promoting the advancement of later cell cycle phases (G2/M)^[35]^. ER+ breast cancer is highly reliant on an intact cyclin D-CDK4/6-Rb axis, as estrogen drives cyclin D1 expression leading to the formation of the cyclin D-CDK4/6 complex, ultimately inducing cell proliferation through the CDK4/6 pathway^[36]^. CDK4/6 inhibitors leverage this by binding the ATP domain of CDK4/6 and halting progression from the G1 to S phase of the cell cycle^[37]^.

The mechanisms of resistance to CDK4/6 inhibitors have yet to be fully elucidated, and in many cases, the clinical relevance of putative mechanisms discovered in preclinical models remains unconfirmed^[38,39]^. Recognized resistance mechanisms include amplification of members of the cyclin D-CDK4/6 axis or downregulation of CDK4/6 repressor proteins, such as p21 and p27, which may thwart the direct effects of these drugs^[38,40-42]^. Additionally, alterations in *RB1*, *FAT1*, or the PI3K/AKT/mTOR and KRAS signaling pathways may act to circumvent the G1/S checkpoint in the presence of CDK4/6 inhibitors, inducing cell cycle progression independent of the cyclin D-CDK4/6 axis^[38,39,43-46]^.

## CIRCULATING CELL-FREE DNA FOR LIQUID BIOPSY AND COMPANION DIAGNOSTICS

In recent years, liquid biopsy approaches have emerged, intending to provide molecular information for biomarker-directed precision oncology from a fluid sample^[24]^. Traditionally, accessing this information has required invasive procedures, like tissue biopsy, which are not always feasible depending on the nature and location of the tumor, as well as the health of the patient. These limitations are particularly relevant for metastatic breast cancer patients, where ER+/HER2- breast cancer recurrences often occur past 5 years. In this clinical setting, the primary tissue sample may not represent metastatic disease, and the metastatic sites are often in areas difficult to biopsy (e.g., bone and lung)^[47,48]^. Here, a liquid biopsy may bypass the procedural, spatial, and temporal obstacles of tissue biopsy by encompassing disease heterogeneity and new metastases while being easily repeated throughout tumor progression and different lines of treatment^[49]^. Therefore, liquid biopsy is advancing as a valuable approach to enable contemporaneous and minimally invasive testing of tumor-specific analytes in cancer patients^[50]^.

For most liquid biopsy applications, the body fluid of choice is peripheral blood plasma. This has previously presented challenges in detecting brain tumors or metastatic sites, likely due to the blood-brain barrier; however, newer liquid biopsy technologies have led to improvements in sensitivity^[51,52]^. Regardless, other accessible fluids may be more informative depending on the tumor type (e.g., saliva for oral cancer, urine for bladder cancer, and cerebrospinal fluid for glioma)^[53]^. There are also a variety of components from the tumor which may be assessed through liquid biopsy, including intact tumor cells (e.g., CTCs), cell-free DNA (cfDNA), extracellular vesicles, cell-free RNA, and more^[24]^. Herein, we focus our discussion on ctDNA as a liquid biopsy biomarker of response to anti-CDK4/6 therapy due to the wealth of recent studies on this topic and the precedent of ctDNA in other settings of precision oncology (e.g., for EGFR tyrosine kinase inhibitors in lung cancer)^[49,54]^. Other liquid biopsy analytes and their role as biomarkers for CDK4/6 inhibitor treatment have been reviewed elsewhere^[23,55,56]^.

ctDNA molecules containing tumor-derived genetic and epigenetic features are released into the bloodstream as tumor cells die^[49]^. ctDNA usually represents a small portion of the total circulating cfDNA pool, where most other cfDNA molecules are derived from cells of hematopoietic origin and alternative tissues^[57-59]^. The release of ctDNA is related to cellular turnover (i.e., many apoptotic and necrotic cells), during which a subset of double-stranded DNA fragments associated with chromatin components enters the extracellular space^[60-62]^. ctDNA levels can be as low as < 0.01% of the entire cfDNA pool, with variations depending on tumor size, stage, anatomical location, treatment, and the biological propensity of tumor cells to release their DNA into the circulation^[51,58,63-65]^. Once in the circulation, clearance of ctDNA by multiple mechanisms (e.g., nuclease-mediated degradation, phagocytosis, and renal excretion) occurs quickly with a half-life between 16 minutes and two hours^[66-68]^. This property enables cfDNA to portray a “real-time” snapshot of the patient’s disease state^[49,69]^.

Analysis of ctDNA through liquid biopsy may be relevant in many clinical stages of CDK4/6 inhibitor treatment, such as prognostication, personalizing therapeutics (e.g., determining which patients should receive anti-CDK4/6 therapy or adding other agents), monitoring treatment response, and identifying resistance [Figure 1]. ctDNA can be analyzed by polymerase chain reaction (PCR)^[70,71]^, which targets a single gene locus, or next-generation sequencing (NGS), which simultaneously profiles dozens or hundreds of genes^[64,72-74]^. Of relevance in ER+/HER2- advanced breast cancer, the *therascreen PIK3CA RGQ PCR kit* detects *PIK3CA* mutations to help direct PI3Ka inhibitor treatment (alpelisib)^[75,76]^. NGS-based liquid biopsy assays often include *PIK3CA *in addition to genes of potential relevance to resistance mechanisms to endocrine therapy, such as *ESR1* and *PTEN*^[77]^. Thus, although not yet established in the CDK4/6 setting, ctDNA shows considerable promise as means of biomarker detection for genotype-guided precision oncology.

## GENETIC-BASED LIQUID BIOPSY BIOMARKERS OF CDK4/6 INHIBITOR EFFICACY AND RESISTANCE

Currently, there are no clinically validated liquid biopsy biomarkers to distinguish patients with differential benefits to anti-CDK4/6 therapy. Here we report the main ctDNA liquid biopsy biomarkers for CDK4/6 inhibitor treatment investigated at baseline or time of progression. The main focus of the studies outlined in this section are alterations of genes related to cell cycle regulation using ctDNA and their relationship to outcomes [Table 1].

**Table 1:** Summary of the main genomic alterations interrogated in ctDNA as biomarkers for CDK4/6 inhibitor resistance

**Genomic alteration**	**ctDNA sample**	**Cohort**	**Prevalence**	**Technique**	**Main findings**	**Reference**
**RB1 **	Baseline	PALOMA-3: Palbociclib plus fulvestrant	27 of 156 patients (17.3%)	Target panel NGS (17 genes)	Patients in the palbociclib treatment arm with loss of *RB1* had worse median PFS compared to wild-type (exact PFS not reported)	O’Leary *et al*.^[78]^
	Baseline	MONALEESA 2,3,7: Ribociclib plus endocrine therapy	26 of 1,534 patients (1.7%)	Target panel NGS (~600 genes)	Patients with* RB1* mutated tumors did not have significantly different median PFS with ribociclib treatment compared to placebo (mutant: 9.2 *vs*. 3.7 months placebo *vs*. ribociclib arm)	Bertucci *et al*.^[79]^
	Progression	Three case reports: Palbociclib plus fulvestrant or ribociclib plus letrozole	NA	Custom library for RB1 and TP53 coding sequence/ Guardant 360 assay (73 genes)	Patients had five different loss-of-function genetic alterations of *RB1* after exposure to CDK4/6 inhibitors and coinciding with resistance	Condorelli *et al*.^[44]^
	Baseline and End-of-treatment	PALOMA-3: Palbociclib plus fulvestrant	Acquired in 6 of 127 patients (4.7%)	Exome sequencing/ Target panel NGS/ ddPCR	Patients exclusively acquired *RB1* alterations in the palbociclib treatment arm	O’Leary *et al*.^[39]^
**ESR1 **	Baseline	PALOMA-3: Palbociclib plus fulvestrant	91 of 360 patients (25.3%)	ddPCR	Patients in the palbociclib treatment arm had similarly improved median PFS regardless of *ESR1* status, although patients in the placebo arm with *ESR1* mutations had worse PFS compared to wild-type (palbociclib arm: 9.4 *vs*. 9.5 months mutant *vs*. wild-type; placebo arm: 3.6 *vs*. 5.4 months mutant *vs*. wild-type)	Fribbens *et al*.^[80]^
	Baseline and End-of-treatment	PALOMA-3: Palbociclib plus fulvestrant	Acquired in 25 of 195 patients (12.8%)	Exome sequencing/ Target panel NGS/ ddPCR	Patients acquired *ESR1 Y537S* mutations in both treatment arms and were associated with improved median PFS compared to patients who did not acquire the mutation (13.7 *vs*. 7.4 months acquired *vs*. not acquired)	O’Leary *et al*.^[39]^
	Baseline	PALOMA-3: Palbociclib plus fulvestrant	72 of 331 patients (21.8%)	Target panel NGS (17 genes)	Patients in the placebo arm with *ESR1* mutations had worse PFS compared to wild-type (exact PFS not reported)	O’Leary *et al*.^[78]^
	Baseline	MONARCH-2: abemaciclib and fulvestrant	147 of 248 patients (59.3%)	ddPCR	Patients in the abemaciclib treatment arm had improved PFS regardless of *ESR1* status but observed a higher numerical median PFS in patients with *ESR1* mutant tumors compared to wild-type (abemaciclib arm: 20.7 *vs*. 15.3 months mutant *vs*. wild-type; placebo arm: 13.1 *vs*. 11.3 months mutant *vs*. wild-type). Patients with *ESR1* mutations also had improved OS (abemaciclib arm: not reached *vs*. 52.2 months mutants *vs*. wild-type; placebo arm: 42.2 *vs*. 29.4 months mutant *vs*. wild-type)	Tolaney *et al*.^[81]^
**PIK3CA**	Baseline	PALOMA-3: Palbociclib plus fulvestrant	129 of 395 patients (33%)	BEAMing assay	Patients in the palbociclib treatment arm had similarly improved PFS regardless of *PIK3CA* status (palbociclib arm: 9.5 *vs*. 9.9 months mutated *vs*. wild-type; placebo arm: 3.6 *vs*. 4.6 months mutated *vs*. wild-type)	Critstofanilli *et al*.^[13]^
	Baseline	MONARCH-2: abemaciclib and fulvestrant	96 of 219 patients (43.8%)	ddPCR	Patients in the abemaciclib treatment arm had similarly improved PFS regardless of *PIK3CA *status, although patients in the placebo treatment arm with *PIK3CA* mutations had worse median PFS compared to wild-type (abemaciclib arm: 17.1 *vs*. 16.9 months mutant *vs*. wild-type; placebo arm: 5.7 *vs*. 12.3 months mutant *vs*. wild-type)	Tolaney *et al*.^[81]^
	Baseline	Palbociclib or ribociclib plus fulvestrant or letrozole	12 of 30 patients (40%)	ddPCR	Patients treated with palbociclib or ribociclib plus endocrine therapy with* PIK3CA* mutations had a worse median PFS compared to wild-type (7.44 *vs*. 12.9 months mutant *vs*. wild-type)	Del Re *et al*.^[82]^
	Baseline	PALOMA-3: Palbociclib plus fulvestrant	55 of 331 patients (16.6%)	Target panel NGS (17 genes)	PIK3CA mutations were not identified as predictive (exact PFS not reported)	O’Leary *et al*.^[78]^
	Baseline and End-of-treatment	PALOMA-3: Palbociclib plus fulvestrant	Acquired in 15 of 195 patients (7.6%)	Exome sequencing/Target panel NGS/ddPCR	Patients acquired *PIK3CA* mutations in both treatment arms and were associated with improved median PFS compared to patients who did not acquire a PIK3CA mutation (12.7 *vs*. 9.2 months acquired *vs*. not acquired)	O’Leary *et al*.^[39]^
	Baseline	MONALEESA-7: Ribociclib plus endocrine therapy	139 of 489 patients (28%)	Target panel NGS (~600 genes)	Patients in the ribociclib treatment arm had improved median PFS compared to the placebo arm, and this was more prominent in patients with wild-type *PIK3CA* compared to *PIK3CA* mutations (ribociclib arm: 14.8 *vs*. 24.7 months mutant *vs*. wild-type; placebo arm 12.9 *vs*. 12.2 months mutant *vs*. wild-type)	Bardia *et al*.^[83]^
**FGFR**	Progression	MONALEESA-2: Ribociclib plus letrozole	20 of 427 patients (5%)	Guardant360 assay (73 genes)	Patients in the ribociclib treatment arm with *FGFR1* alterations had a worse median PFS (10.61 *vs*. 24.84 months mutant *vs*. wild-type), although significance was not achieved due to the small sample size	Formisano *et al*.^[41]^
	Baseline	PALOMA-3: Palbociclib plus fulvestrant	20 of 401 patients (4.9%)	Target panel NGS (17 genes)	Patients with* FGFR1* amplifications had a worse median PFS in both treatment arms (palbociclib arm: 3.9 *vs*. 12 months mutant *vs*. wild-type; placebo arm: 1.8 *vs*. 4.8 months mutant *vs*. wild-type)	O’Leary *et al*.^[78]^
	Baseline and End-of-treatment	PALOMA-3: Palbociclib plus fulvestrant	Acquired in 2 of 195 patients (1%)	Exome sequencing/Target panel NGS/ddPCR	Patients acquired *FGFR2* alterations with no apparent difference between treatment arms.	O’Leary *et al*.^[39]^
**TP53**	Baseline	PALOMA-3: Palbociclib plus fulvestrant	52 of 331patients (15.7%)	Target panel NGS (17 genes)	Patients with *TP53* mutations had a worse median PFS in both treatment arms, and no interaction with treatment was observed (palbociclib arm: 3.7 *vs*. 12.7 months mutant *vs*. wild-type; placebo arm: 1.8 *vs*. 5.4 months mutant *vs*. wild-type)	O’Leary *et al*.^[78]^
	Baseline	MONALEESA-7: Ribociclib plus endocrine therapy	92 of 489 patients (19%)	Target panel NGS (~600 genes)	Patients with *TP53* mutations had a worse median PFS in both treatment groups (ribociclib arm: 9.2 *vs*. 24.7 months mutant *vs*. wild-type; placebo arm: 7.2 *vs*. 13.0 months mutant *vs*. wild-type)	Bardia *et al*.^[83]^
**KRAS**	Baseline and on treatment	Palbociclib plus fulvestrant	66 of 106 patients (62.2%)	ddPCR	Patients treated with palbociclib and fulvestrant with baseline *KRAS* mutations had a worse median PFS compared to wild-type (3 *vs*. 17.8 months mutant *vs*. wild-type)	Raimondi *et al*.^[45]^
**CCND1**	Baseline	MONALEESA-7: Ribociclib plus endocrine therapy	51 of 489 patients (10%)	Target panel NGS (~600 genes)	Patients with *CCND1* alterations had a worse median PFS in both treatment arms and patients with altered *CCND1* also had a significant treatment interaction effect (ribociclib arm: 12.9 *vs*. 22.1 months mutant *vs*. wild-type; placebo arm: 5.5 *vs*. 11.3 months mutant *vs*. wild-type)	Bardia *et al*.^[83]^
**MYC**	Baseline	MONALEESA-7: Ribociclib plus endocrine therapy	35 of 489 patients (7.1%)	Target panel NGS (~600 genes)	Patients with *MYC* alterations had a worse median PFS in both treatment arms (ribociclib arm: 7.3 *vs*. 24.7 months mutant *vs*. wild-type; placebo arm: 7.2 *vs*. 12.9 months mutant *vs*. wild-type)	Bardia *et al*.^[83]^

ctDNA: Circulating tumor DNA; NGS: next-generation sequencing; PFS: progression-free survival; ddPCR: droplet digital polymerase chain reaction; OS: overall survival.

Most studied resistance mechanisms converge on Rb modulation since it is the central target of CDK4/6 to control cell cycle progression^[38]^. Genetic alterations of *RB1* may cause its inactivation and confer resistance to CDK4/6 inhibitors. For example, in the PALOMA-3 trial, loss of *RB1* detected via baseline ctDNA was associated with worse PFS for patients in the palbociclib plus fulvestrant treatment group^[78]^. These results suggest that *RB1* alterations may be prognostic and potentially predictive; however, this study could not determine the treatment interaction effect due to the small sample size. This was further supported by an analysis of ctDNA from patients in the MONALEESA 2, 3, and 7 trials, which found that for patients with *RB1* mutations, ribociclib plus endocrine therapy did not significantly improve median PFS^[79]^. Furthermore, a small clinical report of three patients was the first to identify loss-of-function *RB1 *mutations in ctDNA sampled at the time of acquired CDK4/6 inhibitor resistance^[44]^. This was later supported by an analysis of PALOMA-3 matched baseline and end-of-treatment ctDNA samples, which found loss-of-function *RB1* mutations were exclusively acquired in 4.7% of patients treated with palbociclib plus fulvestrant, suggesting they were selected for in treatment resistance. However, due to the small number of acquired* RB1* mutations, no associations were made to PFS^[39]^. Altogether, these results and biological support of *RB1* loss-of-function as a resistance mechanism suggest that genomic alterations of *RB1* detected in ctDNA may be predictive of CDK4/6 inhibitor resistance. However, the low prevalence of these alterations suggests other resistance mechanisms are involved, and *RB1* mutations may serve as a predictive biomarker of resistance for a limited portion of patients on CDK4/6 inhibitors. 

In addition to *RB1* mutations, genomic alterations in *ESR1* have been investigated due to the ER mitogenic pathway being critical to cyclin D-CDK4/6 dependence and the inactivation having a potential role in resistance to CDK4/6 inhibitors^[46]^. First, multiple analyses of the PALOMA-3 trial revealed that patients with baseline ctDNA *ESR1* mutations had a worse median PFS solely in the placebo treatment arm^[78,80]^. These findings support the role of *ESR1 *mutations as a biomarker of endocrine resistance, and no treatment interaction effect was observed, indicating the lack of predictive potential of *ESR1* mutations for CDK4/6 inhibitor resistance. A later study of PALOMA-3 found that 13 of 195 patients lost *ESR1* mutations between baseline and end-of-treatment, whereas 25 patients in both treatment groups acquired the alteration, suggesting *ESR1 *mutations promote resistance to fulvestrant and do not predict CDK4/6 inhibitor treatment benefit^[39]^. Lastly, a recent analysis of ctDNA from the MONARCH-2 trial found that abemaciclib plus fulvestrant improved PFS regardless of *ESR1* status, but patients with *ESR1* mutations had a higher numerical median PFS in both treatment arms^[81]^. This study also observed an unexpectedly high prevalence of *ESR1* mutations and increased OS for patients with *ESR1* mutations. The contrast of these findings is unclear but may be explained by differences in sample size, patient criteria, and analytical techniques. Altogether, *ESR1 *mutations are not a promising candidate biomarker for CDK4/6 inhibitor resistance and may be more informative for endocrine resistance.

*PIK3CA* mutations have also been interrogated by ctDNA due to their upstream role in cell cycle regulation through the PI3K/AKT/mTOR pathway, which interacts with estrogen receptors and potentially impacts CDK4/6 inhibitor resistance^[38]^. For instance, analyses of the PALOMA-3 trial found that palbociclib plus fulvestrant treatment similarly improved PFS in patients with mutated or wild-type *PIK3CA *in baseline ctDNA, indicating that *PIK3CA* genomic alterations are not predictive of CDK4/6 inhibitor benefit^[13,78]^. In support of this finding, a study of baseline ctDNA from the MONARCH-2 trial found patients in the abemaciclib treatment arm had similarly improved PFS regardless of *PIK3CA* mutation status^[81]^. In addition, patients in the placebo arm with mutant *PIK3CA* had a worse median PFS, suggesting the role of *PIK3CA* mutations in endocrine therapy resistance instead of as a biomarker of CDK4/6 inhibitor treatment. In contrast, another study of baseline ctDNA of advanced breast cancer patients treated with palbociclib or ribociclib found that patients with *PIK3CA* mutations had a shorter median PFS than wild-type^[82]^. Due to a lack of a control treatment arm, this study could not assess the treatment effect with *PIK3CA*. Still, these findings suggest *PIK3CA* mutations may act as a prognostic biomarker for advanced breast cancer patients receiving treatment with CDK4/6 inhibitors.

Furthermore, the lack of predictive potential of *PIK3CA* has been supported in multiple studies. An additional PALOMA-3 analysis of matched baseline and end-of-treatment ctDNA samples found that 7.6% of patients acquired *PIK3CA* mutations across both treatment arms, suggesting that *PIK3CA* mutations emerge due to fulvestrant resistance^[39]^. Interestingly, patients with acquired *PIK3CA* mutations had an improved PFS than those who did not; however, this trend was seen with the acquisition of any new mutation (including *ESR1*), suggesting that novel alterations are more likely to emerge in patients with a longer duration of treatment. Moreover, a recent study analyzing ctDNA from the MONALEESA-7 trial found that patients in the ribociclib treatment arm had an improved median PFS, but this was more pronounced in patients with wild-type *PIK3CA*^[83]^. However, this difference was not statistically significant, reinforcing the limited potential of *PIK3CA* as a predictive biomarker of CDK4/6 inhibitor resistance.

In addition, FGFR genetic alterations have been investigated in plasma ctDNA of patients treated with CDK4/6 inhibitors due to evidence of abnormal FGFR signaling, driving *CCND1* overexpression and MAPK activation, contributing to resistance^[38]^. One analysis of baseline ctDNA from the MONALEESA-2 trial found that for patients in the ribociclib treatment arm, *FGFR1* alterations were related to worse PFS, although significance was not achieved due to the small sample size^[41]^. In further support, a study of ctDNA from the PALOMA-3 trial found that *FGFR1* amplifications were associated with worse PFS in both treatment groups, suggesting that the alteration may be related to endocrine resistance^[78]^. Furthermore, in an assessment of pre- and post-treatment ctDNA samples from PALOMA-3, *FGFR2* was acquired in 2 of 195 patients, with no apparent difference between treatment groups^[39]^. Altogether these findings support FGFR alterations, specifically *FGFR1* amplifications, as a possible prognostic biomarker for patients treated with CDK4/6 inhibitors and endocrine therapy.

*TP53* is a tumor suppressor gene whose product induces antiproliferative factors, such as *CDKN1A*, and its modification in ctDNA has also been investigated in many studies. For example, genomic alterations of *TP53* in ctDNA from the PALOMA-3 trial were assessed, revealing that patients with *TP53* mutations had significantly worse PFS in both treatment arms, and no interaction with treatment was observed^[78]^. This work also found that *TP53* mutations were connected to a distinct aggressive phenotype with more metastases but could not rule out the presence of these mutations due to clonal hematopoiesis. Moreover, a MONALEESA-7 biomarker analysis found that *TP53* mutations in baseline ctDNA were associated with progression independently from treatment with ribociclib and did not predict response to therapy^[83]^. Overall, these studies support *TP53* alterations as prognostic biomarkers and suggest they are not candidate predictive biomarkers of anti-CDK4/6 therapy.

In addition, genomic alterations of *KRAS* in ctDNA have recently been interrogated due to its role upstream of CDK4/6, transducing mitogenic signaling and affecting cyclin D1^[38]^. One study investigated *KRAS* mutations in ctDNA of 106 HR+/HER2- metastatic breast cancer patients treated with palbociclib plus fulvestrant and found that after 18 months, all patients with *KRAS* alterations had progressive disease^[45]^. In contrast, only one *KRAS* wild-type patient had progressed. Accordingly, patients with mutated *KRAS* had a worse median PFS compared to wild-type, supporting the potential of *KRAS* mutations as a prognostic biomarker. Further study with a control treatment group is required to determine the treatment interaction effect before conclusions can be made. For instance, previous studies on ctDNA from the PALOMA-3 trial have investigated *KRAS* mutations, but likely due to the low frequency of aberrations, have not made associations with PFS^[39,78]^.

Many other genomic alterations have been investigated in plasma ctDNA of patients treated with CDK4/6 inhibitors due to their role around the cyclin D-CDK4/6-Rb axis or in breast cancer in general, namely* CCND1*, *CDK4*, *CDK6*, *CDKN1*, *CDKN2*, *NF1*, *ERBB2*, *AKT1*, *NRAS*, *HRAS*, *GATA3*, and *MYC*^[39,78,83]^. In particular, a recent study of the MONALEESA-7 samples found that patients with *CCND1* alterations in baseline ctDNA had a worse median PFS for both treatment arms, indicating the role of *CCND1* as a prognostic biomarker^[83]^. The benefit of ribociclib was also greater in patients with *CCND1* altered ctDNA, supporting *CCND1* as a candidate predictive biomarker. Alterations in *MYC* have also been reported as a potential prognostic biomarker for patients treated with CDK4/6 inhibitors in combination with or solo endocrine therapy^[83]^. Otherwise, associations with PFS and treatment have not been reported for many of the above alterations.

In summary, current work suggests *ESR1* and *PIK3CA* mutations in ctDNA have limited CDK4/6 inhibitor biomarker potential, which may be clouded due to implications with endocrine resistance. Alterations in *FGFR1*, *TP53*, and *MYC* may also be prognostic biomarkers of resistance to anti-CDK4/6 therapy, whereas *RB1*, *KRAS,* and *CCND1* may be prognostic and putative predictive biomarkers of CDK4/6 inhibitor resistance. Although numerous potential biomarkers have been identified, many studies have not confirmed the effect of interaction with treatment, and further validation is needed. While considering this, the evidence so far indicates that no singular genetic alteration will serve as an ideal prognostic or significantly predictive biomarker for CDK4/6 inhibitor efficacy or resistance. While some potential predictive biomarkers, such as *RB1* alterations, seem promising, their low prevalence indicates multifactorial resistance mechanisms. As such, analyses that only evaluate one genomic alteration are limited in assessing whether other alterations add predictive value. To assess the vast landscape of CDK4/6 inhibitor resistance mechanisms, future studies should consider a broader range of genomic alterations in ctDNA or, as discussed in later sections, expand to longitudinal monitoring to assess ctDNA dynamics or investigate epigenetic-based features.

## DYNAMIC ctDNA BIOMARKERS OF CDK4/6 INHIBITOR EFFICACY AND RESISTANCE

Monitoring changes in ctDNA levels throughout treatment may reveal more prognostic and predictive information than a single time point measurement. Serial monitoring of ctDNA levels could provide a dynamic biomarker that allows personalized modifications to treatment, such as adding or switching to a more effective therapy for non-responsive patients^[84]^. In investigations of the utility of dynamic liquid biopsy biomarkers, questions remain concerning sampling time points, assays used, and change thresholds. This section discusses research on dynamic ctDNA biomarkers relevant to CDK4/6 inhibitor treatment [Table 2].

**Table 2:** Summary of dynamic ctDNA biomarkers investigated in the CDK4/6 inhibitor treatment setting

**Technique**	**ctDNA sample time points**	**Genetic alteration**	**Cohort**	**Metric**	**Main findings**	**Reference**
ddPCR	Baseline, cycle 1 day 15, and progression	*ESR1* and *PIK3CA*	PALOMA-3: Palbociclib plus fulvestrant	High and low CDR15 based on a threshold determined by Harrell’s c-index and Benjamini-Hochberg p-value corrections	All patients in the palbociclib treatment arm had a CDR15 less than one. For *PIK3CA*, patients with a high CDR15 had a worse median PFS than those with a low CDR15 (4.1 *vs*. 11.2 months high *vs*. low)	O’Leary *et al*.^[85]^
ddPCR	Baseline, day 15, day 30, and progression	*ESR1*	ALCINA: Palbociclib plus fulvestrant	Ratio relative to baseline (mutant copies/mL)	All patients experienced a decrease in ctDNA on day 15 relative to baseline. Patients with early progression had increased ctDNA on day 30, and patients with longer PFS had lower or consistent ctDNA levels relative to baseline. *ESR1* mutations ctDNA on day 30, as opposed to ctDNA clearance, was correlated with worse PFS	Jeannot *et al.*^[86]^
ddPCR monitoring one tumor-specific mutation per patient	Baseline, day 15, day 30 and progression	*PIK3CA* (*n* = 21), *TP53* (*n* = 2), or *AKT1* (*n* = 2)	ALCINA: Palbociclib plus fulvestrant	Ratio relative to baseline (mutant copies/mL)	All patients had a decrease in ctDNA on day 15, but this was not associated with PFS. Patients with undetectable ctDNA on day 30 had an improved PFS compared to those with detectable ctDNA (25 *vs*. 3 months undetectable *vs*. detectable, respectively). ctDNA ratios (day 30/baseline) greater than or less than one were significantly associated with PFS	Darrigues *et al.*^[87]^
Guardant360 assay	Baseline, four weeks	73 genes	Palbociclib or ribociclib plus endocrine therapy	mVAFR for the 79 mutations found between baseline and week 4 assess in groups of high, medium, and low mVAFR groups and as a continuous variable	mVAFR was significantly associated with PFS, whereas single timepoint mean VAFs or absolute changes in mean VAF were not. Patients with high mVAFR had a worse median PFS than those with low mVAFR (4.2 months *vs*. not reached high *vs*. low)	Martinez-Saez *et al.*^[88]^
mFAST-seq	Various	Aneuploidy	CDK4/6 inhibitor plus endocrine therapy	z-score trajectories	Raised z-score trajectories were significantly related to worse PFS, whereas baseline z-scores were not predictive of progression. A single z-score increased in a consecutive blood sample at any follow-up point was not associated with PFS	Dandachi *et al.*^[89]^

ctDNA: Circulating tumor DNA; ddPCR: droplet digital polymerase chain reaction; CDR15: circulating DNA ratio at cycle one day 15 compared to baseline; PFS: progression-free survival; mVAFR: mean variant allele fraction ratio; mFAST-seq: modified Fast Aneuploidy Screening Test-Sequencing System.

Dynamic ctDNA levels in response to CDK4/6 inhibitor treatment were first investigated by monitoring *PIK3CA* and *ESR1* mutant ctDNA from the PALOMA-3 trial collected at baseline, cycle one day 15, and progression^[85]^. *PIK3CA* and *ESR1* mutations were detected in 100 and 114 of 455 baseline samples (22% and 25.6%), with 73 and 65 matched day 15 samples, respectively. For both *PIK3CA* and *ESR1*, a significant decline in ctDNA occurred on day 15, with a more apparent decrease in mutant *PIK3CA* ctDNA in the palbociclib arm and mutant *ESR1* ctDNA in the placebo arm. This group also defined a circulating DNA ratio (CDR15) as the concentration of ctDNA on day 15 compared to baseline. They found that all patients on palbociclib had a CDR15 less than one, possibly due to the cytostatic effect of CDK4/6 inhibitors. For *PIK3CA*, patients with CDR15 above the median had a worse PFS than those below the median; however, this was not seen for *ESR1*. Using an optimal threshold determined by Harrell’s c-index and Benjamini-Hochberg p-value corrections, they found that patients with a high *PIK3CA* CDR15 had an inferior median PFS than those with a low CDR15. Ultimately, this study determined that relative change in ctDNA based on commonly truncal* PIK3CA* mutations was predictive of PFS for patients treated with palbociclib and fulvestrant. Alternatively, ctDNA dynamics based on *ESR1* mutations, which are generally subclonal due to selection of prior endocrine therapy, were not predictive of clinical outcome. Altogether, these results indicate that early evaluation of ctDNA dynamics with truncal mutations may be a predictive biomarker of PFS for patients on CDK4/6 inhibitor treatment.

In addition, a subsequent study also assessed *ESR1* mutations longitudinally in the ACLINA cohort of 59 ER+/HER2- metastatic breast cancer patients treated with palbociclib plus fulvestrant^[86]^. They found *ESR1* mutations in 28.8% of the baseline samples, but these were not associated with PFS. In addition, they found that all patients experienced a decrease in ctDNA on day 15 relative to baseline. In contrast, on day 30, patients with early progression had increased ctDNA, and patients with longer PFS had lower or consistent ctDNA levels. They found that the presence of *ESR1* mutant ctDNA on day 30, as opposed to ctDNA clearance, was correlated with worse PFS, suggesting the potential of ctDNA detection on day 30 of treatment as a prognostic biomarker.

Further support for ctDNA ratios on day 30 as a biomarker comes from additional analysis of ctDNA from patients in the ALCINA cohort^[87]^. First, this study used archived tumor tissue to identify trackable mutations based on a panel of 15 driver genes. Next, they assessed serial plasma samples for 25 patients with either *PIK3CA*, *TP53*, or *AKT1* mutations and found that baseline ctDNA levels had no association with PFS. In addition, they found that all patients had a decrease in ctDNA on day 15, which was not associated with PFS. In contrast, three kinetic patterns appeared on day 30, with nine patients displaying a continuous decreased or undetected ctDNA, one patient with consistent ctDNA levels, and five patients with an increase in ctDNA. Patients with undetectable ctDNA on day 30 had an improved PFS compared to those with detectable ctDNA. Furthermore, the radiological response had high concordance with ctDNA detection, with general decreases or undetectable ctDNA for non-progressive disease compared to rising ctDNA between days 15 and 30 for progressive disease. However, this study assessed concentrations of ctDNA with respect to disease progression and found overlap in progressive versus non-progressive disease at all time points, highlighting the challenge of absolute abundance as a biomarker. Therefore, they assessed ctDNA ratios relative to baseline and found low ratios on day 15, with no significant difference in decline between patients with and without disease progression. Instead, they found that ctDNA ratios on day 30 relative to baseline distinguished patients with non-progressive disease (ratio < 1) and progressive disease (ratio > 1) and that high ctDNA ratios were associated with worse PFS. This study supports that monitoring of ctDNA is related to radiological progression and demonstrates the potential of ctDNA dynamics on day 30 relative to baseline as a prognostic biomarker for patients treated with CDK4/6 inhibitors.

In contrast to the studies highlighted above, ctDNA dynamics can be monitored using the combined signal from profiling changes in many mutated genes instead of specific mutations. For instance, a recent study assessed ctDNA levels at baseline and four weeks for 45 patients treated with CDK4/6 inhibitors and endocrine therapy using the 73-gene Guardant360 assay^[88]^. This work defined a mean variant allele fraction ratio (mVAFR) as an average of mutations found between baseline and week 4 for each patient. They found that mVAFR was significantly associated with PFS, whereas single timepoint mean VAFs or absolute changes in mean VAF were not. One consideration of this study is that they assessed PFS with respect to three mVAFR groups (high, medium, and low) with cutoffs based on their cohort before assessing mVAFR as a continuous variable. Another limitation of this study was that they could not distinguish mutations from clonal hematopoiesis. Altogether, this study illustrates that early ctDNA dynamics in multiple genes may act as a biomarker to identify patients who are likely to progress on CDK4/6 inhibitors and endocrine therapy, which may provide the opportunity to modify or add treatments early on.

An additional study evaluated ctDNA using an untargeted sequencing technique, modified Fast Aneuploidy Screening Test-Sequencing System (mFAST-seq) in longitudinal samples from 49 HR+/HER2- metastatic breast cancer patients treated with CDK4/6 inhibitors^[89]^. In this work, associations between z-score measurements, which are surrogate measurements to ctDNA fraction, were made to clinical outcomes using joint models, which link data over a protracted period of time and time-to-event. They found that raised z-score trajectories were significantly related to worse PFS, whereas baseline z-scores were not predictive of progression. Interestingly, they found that a single rise z-score in a consecutive blood sample at any follow-up point was not associated with PFS. This study highlights the use of different assays in dynamic monitoring and reinforces that trajectories, as opposed to single time points, may be useful biomarkers of progression on anti-CDK4/6 therapy. A limitation of this approach is that mutations were not assessed, which may be relevant in the future for potential interventions or modifications to treatments. Also, this study evaluated z-score measurements at 181 time points for 49 patients, but these were not standardized and did not address the optimal time to sample. They also observed that some patients with progressive disease did not have raised z-scores over time and may be due to long intervals in sampling failing to detect an increase. This may be elucidated by shorter and more consistent sampling in future studies.

Overall, the current literature on monitoring ctDNA dynamics for patients treated with CDK4/6 inhibitors is limited. Differences in methods, patient populations, sampling time points, assays, and change thresholds lead to discordance across studies. Determining ideal change thresholds and timepoints will be essential for downstream clinical decisions. Future research should evaluate a more comprehensive range of consistent time points. Another important consideration is the way mutations are interrogated in ctDNA. Methods can vary dramatically by the number of tumor-specific markers assessed. ddPCR and related single-locus approaches can have high analytical sensitivity and specificity for a specific mutation, but other mutations and subclones that may be relevant to treatment resistance are ignored. Broader targeted panel sequencing approaches can be more robust by simultaneously interrogating multiple mutations and accounting for potential mechanisms of resistance^[64,72]^. Bespoke assays designed for each patient based on tumor tissue sequencing results [e.g., TARgeted DIgital Sequencing (TARDIS) and Signatera] show promise for sensitive and specific ctDNA detection^[63,73,90,91]^, although emergent subclones that are not present in the tissue specimen can be missed^[54]^. Future studies may benefit from combining these approaches to achieve high analytical performance while enabling broad discovery.

## EPIGENETIC-BASED LIQUID BIOPSY BIOMARKERS OF CDK4/6 INHIBITOR EFFICACY AND RESISTANCE

As outlined in the previous sections, most CDK4/6 inhibitor ctDNA studies have focused on genetic alterations (e.g., small nucleotide variants, copy number aberrations, *etc*.). In contrast, epigenetic alterations have been relatively understudied in this context. Emerging methodologies now make it easier to profile epigenetic aberrations in ctDNA, including DNA methylation, fragmentation, and histone modifications^[92]^. This new generation of liquid biopsy investigations has expanded the potential diagnostic use of cfDNA compared to genetic alterations on their own (e.g., providing information on the tissue of origin). Investigating epigenetic-based features has also expanded the number of cfDNA fragments of interest beyond solely mutated tumor-derived fragments. Supporting these new methods is a maturing research infrastructure (e.g., bioinformatics infrastructure, machine learning tools) for handling increasingly complex cancer liquid biopsy data^[93]^. Since genetic alterations in ctDNA have failed to identify clear predictive biomarkers of CDK4/6 inhibitor efficacy and resistance, there is interest in exploring the potential value of epigenetic-based biomarkers in this setting.

### Phenotype of CDK4/6 inhibitor resistance and gene expression profiling

Support for epigenetic liquid biopsy approaches comes from previous work highlighting that phenotypic biomarkers that reflect transcriptomic programs are likely to predict response to CDK4/6 inhibitors. As summarized below, multiple gene expression analyses have demonstrated the effect of CDK4/6 inhibitors on proliferation and cell cycle genes, and patterns associated with resistance have been proposed.

An analysis of ER+/HER2- breast cancer patients in the NeoPalAna trial explored gene expression changes through serial tissue biopsies at baseline, cycle one day 1, cycle one day 15, and surgery. High expression of *CCNE1*, *CCND3*, and *CDKN2D* at cycle one day 15 was associated with resistance to neoadjuvant palbociclib plus anastrozole but not anastrozole alone^[94]^. These gene expression changes suggest that resistant tumors have continual E2F1 activity. Similarly, a gene expression analysis on tissue samples from baseline and surgery from patients treated with preoperative palbociclib found large-scale changes in genes related to proliferation and cell cycle after treatment, including a significant decrease in *CCNE2* expression in antiproliferative responders compared to nonresponders^[95]^.

A substudy of the PALOMA-3 trial yielded partially confirmatory findings^[96]^. Gene expression of 2534 cancer-related genes from 302 patient tumors revealed that high expression of *CCNE1 *- but not other genes related to cell cycle regulation (*CDK4*, *CDK6*, *CCND1,* and *RB1*) - was associated with resistance to palbociclib (median PFS palbociclib arm: 7.6 *vs*. 14.1 months high *vs*. low; placebo arm: 4.0 *vs*. 4.8 months high *vs*. low). The increased predictive power of *CCNE1* mRNA in metastatic biopsies suggests that sampling closer to treatment allows improved identification of predictive biomarkers, which may be facilitated by liquid biopsy. The authors also confirmed the potential role of *CCNE1* as a predictive biomarker in an independent validation cohort of breast cancer patients from the preoperative palbociclib study, where high *CCNE1* levels were associated with a decreased antiproliferative effect with palbociclib.

Further support for *CCNE1 *mRNA as a predictive biomarker stems from a series of studies examining expression levels relative to those of *RB1*. One study of ER+/HER2- preclinical models found that joint decreased expression of *RB1* and increased expression of *CCNE1* commonly occurred at resistance^[97]^. The ratio of *CCNE1* to *RB1 *was then confirmed to be associated with palbociclib resistance among patients in the NeoPalAna trial. Another study of patients treated with abemaciclib and anastrozole alone or combined within the neoMONARCH trial found that resistant tumors had higher expression levels of *CCNE1* and lower levels of *RB1*^[98]^. High tumor *CCNE1 *expression was again associated with poor PFS among 391 patients treated with letrozole plus ribociclib in the MONALEESA-2 trial^[41]^; interestingly, this study also identified *FGFR1 *expression as a putative biomarker for CDK4/6 inhibitors (PFS of 22 months *vs*. not reached for patients with high *vs*. low *FGFR1* expression, respectively).

One limitation of many studies correlating gene expression and PFS is that high and low expression thresholds are often determined above and below the median expression level in the cohort. Thresholds that are biased to the study cohort make it challenging to assess the prognostic or predictive potential, make comparisons across studies, and translate biomarker development to the clinic. Regardless, the current evidence supports that gene expression may predict CDK4/6 inhibitor efficacy or resistance. Considering gene signature assays can become routine in the clinical management of ER+ breast cancer patients, such as MammaPrint and OncotypeDx, specific gene signatures that are predictive of therapy with CDK4/6 inhibitors may be defined and developed^[27,29]^. However, these assays require tissue samples, which have barriers to accessibility and are often limited by the quality and quantity of RNA after FFPE chemical degradation^[99]^. This becomes especially difficult to derive from archival tissue specimens collected years before metastatic relapse. Many of these obstacles may be overcome by assessing transcriptional and epigenetic profiles with liquid biopsy.

### Opportunities of non-mutational signatures of cfDNA

The potential of epigenetic mechanisms and biomarkers for CDK4/6 inhibitor resistance has so far not been examined in detail, and investigating these avenues may provide novel insight. For instance, one recent study showed that treatment with CDK4/6 inhibitors in ER+ breast cancer causes extensive enhancer activation through activator protein-1 (AP-1) transcriptional changes. They found that the widespread chromatin remodeling with CDK4/6 inhibitor treatment may explain the effects of these drugs beyond cell cycle arrest and may be involved in early adaptations leading to resistance^[100]^. These epigenomic changes may also be inferred from various features of ctDNA, such as methylation, fragmentation patterns, and histone modifications [Figure 1].

DNA methylation, namely 5-methylcytosines at CpG sites, is a vital part of cell-type-specific transcriptional regulation, and methylation profiles differ between tumor and normal tissues^[101]^. These differential methylation patterns are maintained in plasma cfDNA and can classify cancer types with high sensitivity in both early and late-stage disease^[102,103]^. Differential plasma cfDNA methylation patterns can also be leveraged to delineate the contribution of various tissues to the cfDNA pool and infer the expression of genes implicated in cancer^[57,103,104]^. One study investigated methylation-based biomarkers in the context of CDK4/6 inhibitors by assessing the methylation status of *ESR1* in plasma cfDNA from a cohort of 49 HR+/HER2- metastatic breast cancer patients treated mostly with endocrine therapy and CDK4/6 inhibitors. Using samples from baseline and at three months, they assessed methylation levels at two main promoters with methylation-specific ddPCR. They found that a greater than 2-fold increase in promoter B or both promoters was associated with a worse prognosis^[105]^. While this study has various limitations, such as a small heterogeneous cohort, inability to dissect endocrine and CDK4/6 inhibitor effects, and analysis of limited loci, it paves the way for both epigenetic and dynamic cfDNA biomarker discovery approaches.

Future studies of DNA methylation in the CDK4/6 inhibitor treatment setting should consider other technical approaches. For instance, while the analysis of limited CpG sites is practical in many clinical settings for simplicity of interpretation and lower costs, expanding to genome-wide explorations may uncover novel candidate resistance biomarkers. Furthermore, bisulfite conversion is necessary for most methylation techniques but can result in excessive loss of DNA due to degradation, which is a substantial challenge for low-input cfDNA samples^[106]^. Forthcoming research should leverage other methods that surpass these limitations by enriching specifically for methylated fragments of cfDNA before sequencing^[104,107,108]^. Moreover, hydroxymethylation is a related epigenetic modification produced by TET enzymes during cytosine demethylation and acts as a marker of active promoters^[109]^. Though less studied in cfDNA, similar enrichment techniques have been developed to assess regions with 5-hydroxymethyl cytosines^[110,111]^. These approaches may be especially useful for revealing surrogates of gene expression that could confer sensitivity or resistance to CDK4/6 inhibitors.

Phenotypic information can also be inferred from distinct fragmentation patterns between ctDNA and other sources of cfDNA. The fragmentation of cfDNA is a non-random process associated with chromatin structure, gene expression, and nuclease content. Differences in nucleosome occupancy patterns at open versus closed chromatin and across varying gene expression levels affect where nucleases can access and fragment the DNA^[92]^. This, in turn, is reflected in the physical characteristics of the cfDNA fragments and their distribution over the genome (i.e., fragmentation features), revealing information about cell and tissue of origin. Early work on cfDNA fragments revealed differences in fragment length, a phenomenon that multiple studies have leveraged to enrich ctDNA and improve the accuracy of cancer detection^[112-117]^.

Beyond fragment length, many other fragmentation features have also been investigated, including relative sequence coverage^[118-124]^, end motifs^[125-129]^, and more^[130-132]^. There is substantial evidence that these features convey information about DNA protection from digestion, which multiple studies have used to create cfDNA deduced nucleosome maps^[58,118,120,133]^. The fragmentation profiles from this work have been correlated with gene expression profiles and permitted identification of cancer type^[118,120,123]^.

The association of fragmentation profiles with transcriptional activity may present opportunities to infer existing candidate gene expression biomarkers of CDK4/6 inhibitor resistance (e.g., *CCNE1* expression) with cfDNA in blood plasma while simultaneously permitting assessment of clinically actionable mutations to direct subsequent therapies. While promising, there remain several practical hurdles to implementing such biomarkers in the clinic. Pre-analytical variables could influence fragmentation features, and their extraction from sequencing data requires complex bioinformatics analysis. However, if these hurdles can be overcome, fragmentation-based biomarkers could greatly extend the utility of ctDNA analysis in the CDK4/6 inhibitor biomarker setting and beyond.

Another class of epigenetic modification has recently been proposed for liquid biopsy applications: post-translational modifications of nucleosomal histones in circulation. These modifications differ in euchromatin compared to heterochromatin and may signal transcriptionally active (e.g., H3K4me3, H3K36me3) or repressed (e.g., H3K27me3, H3K9me3) regions of the genome^[134]^. Furthermore, histone modifications may signal chromatin remodeling and transcriptional changes between cancer and healthy cells. Initial work found a global decrease in repressive histone markers across multiple cancer types^[135,136]^. In a recent study, cell-free chromatin immunoprecipitation and sequencing (ChIP-seq) was conducted to identify regions associated with transcriptionally active histone modifications^[137]^. This approach revealed signals reflective of distinct tissues and cancer types, potentially expanding the toolbox for biomarker discovery in the context of CDK4/6 inhibitors for breast cancer patients.

Overall, epigenetic profiling of cfDNA has many potential benefits that have gone mostly unexplored in the context of breast cancer resistance to CDK4/6 inhibitors. More investigation is needed to elucidate epigenetic signatures and determine which features are the most informative from the vast list growing in the literature. A combination of approaches may increase the predictive power compared to any method alone and increase the ability to direct subsequent therapies^[138-140]^.

## CONCLUSION

Biomarkers are essential for precision oncology, and despite widespread research efforts, no clinically validated biomarkers beyond breast cancer subtype have been established to guide the use of CDK4/6 inhibitors. Liquid biopsy presents many benefits for biomarker development due to being minimally invasive and encompassing the heterogeneity of metastatic sites. Accordingly, putative predictive biomarkers of resistance to treatment with CDK4/6 inhibitors have been found in ctDNA such as *RB1*, *KRAS*, and *CCND1* genomic alterations; dynamic changes in ctDNA, such as changes in truncal mutations between baseline and cycle one day 15 or 30, or ctDNA clearance on day 30. Expression of E2F target genes such as *CCNE1 *is also associated with resistance and could someday be reflected through emerging epigenetic ctDNA analysis methodologies. In addition, prognostic biomarkers such as *ESR1*, *PIK3CA*, *FGFR1*, *TP53*, and *MYC* alterations have also been identified.

To advance further biomarker discovery, future studies of trials or cohorts that included a control arm (e.g., endocrine therapy alone) will be especially valuable to permit the assessment of treatment interaction effects. In addition, it would be beneficial to investigate a broader range of alterations within ctDNA, given the low likelihood that a single alteration will be an ideal biomarker to the vast mechanisms of resistance. Thresholds for expression or dynamic changes in ctDNA should also be determined independently from the patient cohort in which they are studied to increase generalizability and reproducibility. Moreover, future work on ctDNA dynamics should sample a larger range of time points. There is also enormous potential for phenotype-based biomarkers, which may reflect widespread epigenetic changes and inform changes in tumor biology associated with resistance. There is a lack of established mutation-based liquid biopsy biomarkers, and features of cfDNA related to methylation, fragmentation, and histone modifications are uncharted in the context of CDK4/6 inhibitor resistance. These ctDNA epigenomic profiles should be leveraged for new biomarker discovery. Although this review has focused on the ER+/HER2- subtype of breast cancer, it is conceivable that novel predictive biomarkers could identify other patients likely to respond. Lastly, there have been substantial investigations into novel targeted agents for treatment after CDK4/6 inhibitor resistance. For instance, preclinical and clinical data indicate that treatment with novel endocrine therapies, PI3K/MAPK pathway inhibitors, downstream mitotic kinase inhibitors, and DNA-damage related inhibitors may each have a role in the treatment of CDK4/6 inhibitor-resistant disease^[46]^. With further development, liquid biopsy biomarkers may help direct these subsequent therapies in the future, increasing the likelihood of successful clinical development and the potential impact on patient outcomes.

## DECLARATIONS

### Copyright

© The Author(s) 2022.
